# Autologous rectus abdominis fascia sling surgery following unsuccessful synthetic midurethral sling

**DOI:** 10.1002/iju5.12719

**Published:** 2024-03-11

**Authors:** Yoshitaka Kurano, Nobutaka Shimizu, Rie Yoshimura, Ryohei Iga, Kaya Atagi, Tomoya Nao, Hideo Fukuhara, Satoshi Fukata, Shingo Ashida, Keiji Inoue

**Affiliations:** ^1^ Department of Urology, Kochi Medical School Kochi University Nankoku Japan; ^2^ Pelvic Floor Center, Kochi Medical School Kochi University Nankoku Japan

**Keywords:** fascia, internal urethral sphincter, midurethral sling, rectus abdominis, urinary stress incontinence

## Abstract

**Introduction:**

We performed autologous rectus abdominis fascia sling surgery using Advantage™ following an unsuccessful synthetic midurethral sling.

**Case presentation:**

At the age of 76 years, the patient experienced stress urinary incontinence recurrence. A 1‐h pad test resulted in 259 g of leakage. A pressure flow study verified urine leakage while coughing and straining without detrusor overactivity. Abdominal leak point pressure was 10 cmH_2_O. Autologous rectus abdominis fascia sling surgery was performed using Advantage™. One month postoperatively, a 1‐h pad test resulted in 0 g of leakage.

**Conclusion:**

We believe that this method will allow the fascia sling procedure to be performed reliably even if one is unfamiliar with conventional autologous rectus abdominis fascia sling surgery.

**Keynote message**

With the advent of tension‐free vaginal tape or transobturator tape as the prevailing approach for stress urinary incontinence, the number of clinicians with expertise in autologous rectus abdominis fascia sling surgery has markedly diminished. We are confident that the approach we present will enable the execution of the autologous rectus abdominis fascia sling procedure even if prior knowledge of this conventional procedure is lacking.

**Abbreviations & Acronyms**
ALPP: abdominal leak point pressure
FDV: first desire to void
MUS: midurethral sling
NDV: normal desire to void
PFS: pressure flow study
SDV: strong desire to void
SUI: stress urinary incontinence
TVT: tension‐free vaginal tape

## Introduction

The management of female patients who experience recurrent SUI following an initially failed mesh‐based MUS procedure is a topic of ongoing clinical discussion. The literature outlines many treatment approaches, including injectable therapy, repeated synthetic MUS, and retropubic slings that utilize either mesh or autologous fascia.^1^ Here, we performed autologous rectus abdominis fascia sling surgery using Advantage™ following unsuccessful synthetic MUS.

## Case presentation

A 69‐year‐old woman diagnosed with SUI was admitted to our facility. We performed a midurethral tissue fixation system sling surgery for SUI.^2^ At the age of 73, the patient had a recurrence of SUI and underwent MUS surgery using TVT after the removal of the previous sling tape. Subsequently, at the age of 76 years, the patient experienced SUI recurrence.

A 1‐h pad test resulted in 259 g of leakage. A PFS verified urine leakage while coughing and straining without detrusor overactivity. The FDV was at 62 mL, NDV was at 113 mL, SDV was at 166 mL, and ALPP was at 10 cmH_2_O. No obstructive pattern was observed. The results of the urinary storage function indicated mild hypersensitivity. Because of severe intrinsic sphincter deficiency, we performed autologous rectus abdominis fascia sling surgery using Advantage™.

**Fig. 1:**
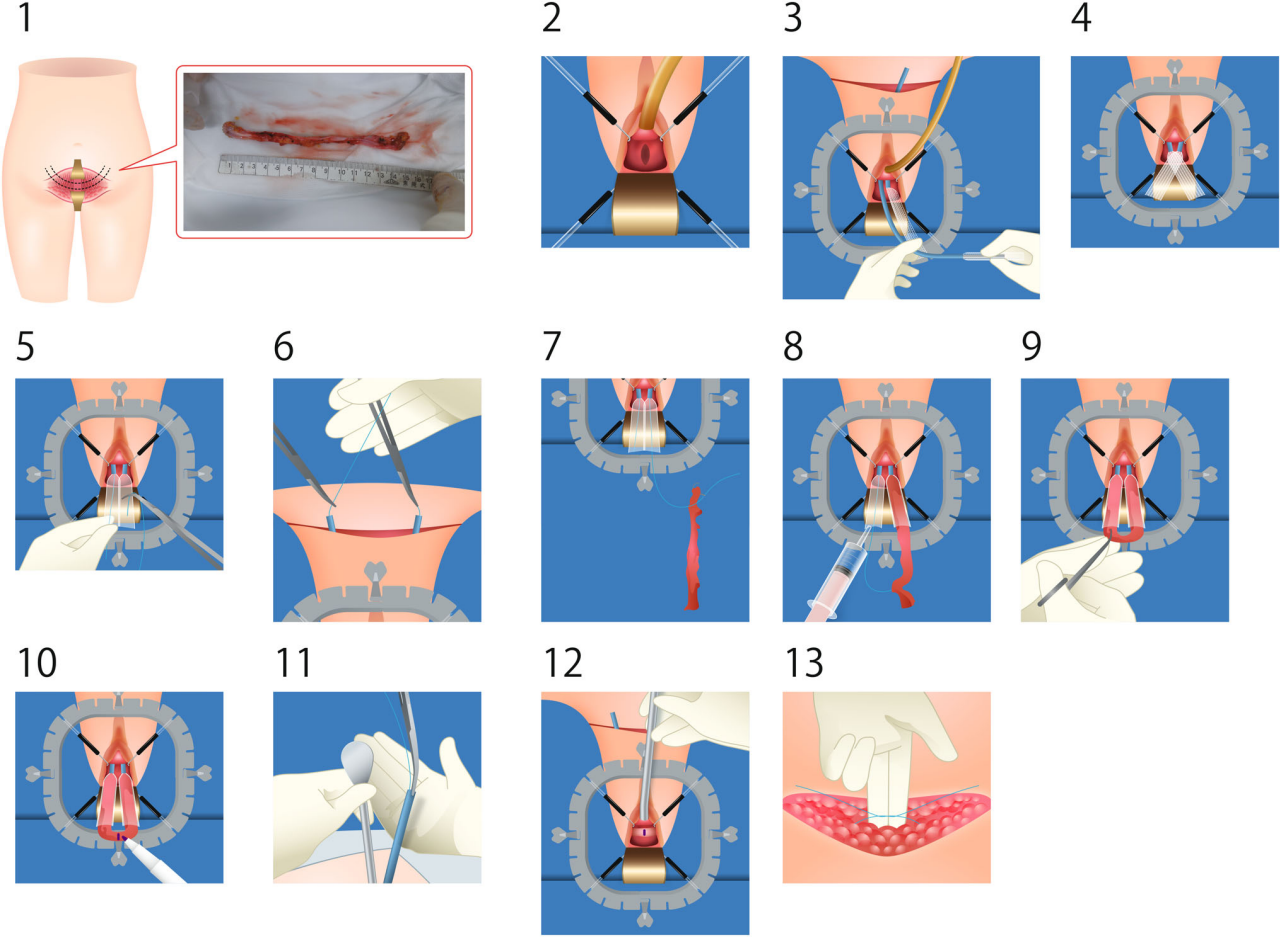
Surgical procedure.

The patient was operated on in the lithotomy position under general anesthesia. A lower abdominal incision was made through a Pfannenstiel incision, and a 2 × 15 cm rectus abdominis fascia was harvested. The lower abdominal wound was excised at the end of the procedure. The gathered fascia was encased in sterile gauze and preserved until insertion procedure (Fig. 1‐1). After insertion of the urinary catheter, hydrodissection of the mid to distal anterior vaginal wall was performed to provide space for Advantage Fit™ to pass through (Fig. 1‐2). First, the previously implanted TVT mesh tape was resected as much as possible. The Advantage Fit™ needle was advanced along the posterior pubic surface, the rectus abdominis muscle was punctured, the bilateral lower abdomen was passed, and cystoscopy was performed to confirm the absence of accidental puncture of the bladder. The sleeves were cut to a length of approximately 8 cm on each side, and the TVT was extracted (Fig. 1‐3, ‐4). From the lower abdominal side, we passed a nonabsorbable nylon thread through the dilator tube lumen and withdrew it from the sleeve side (Fig. 1‐5, ‐6). The thread was tied to both ends of the harvested fascia and pulled upward; the fascia was then inserted into a sleeve (Fig. 1‐7, ‐8, ‐9). Marking the midline of the fascia is important for accurate placement (Fig. 1‐10). The urinary catheter was removed and pulled down with a 23‐Fr urethral bougie to keep the fascia close to the middle of the urethra while pulling out the dilator tube or sleeves to provide sufficient tension to the fascia without closing the urethral lumen (Fig. 1‐11, ‐12). The bilateral nylon threads tied to the fascia were ligated onto the surface of the rectus abdominis muscle, with sufficient room for two fingers between the threads and the rectus abdominis muscle. We closed the vaginal wall and lower abdomen and terminated the operation (Fig. 1‐13).

The operative time was 140 min. Estimated blood loss was scant. No perioperative transfusions, infections, or other complications were observed. The urinary catheter was removed 3 days postoperatively, and transabdominal echo confirmed the absence of residual urine on postoperative day 5. The patient was discharged on postoperative day 6.

One month postoperatively, a 1‐h pad test resulted in 0 g of leakage. PFS verified the absence of urine leakage during coughing. The FDV was at 198 mL, NDV was at 260 mL, and SDV was at 385 mL. No obstructive pattern was observed.

## Discussion

The failure rate of synthetic MUS ranges from 12% to 20%. Persistent or recurrent incontinence as well as recurrent incontinence following sling excision due to complications such as erosion, pain, and retention are potential causes of sling failure.^3^ Studies comparing primary and repeat synthetic slings have found that patients who use repeat synthetic slings experience a decreased subjective cure rate compared with that in those who use primary slings.^4^, ^5^, ^6^, ^7^

Urologists have traditionally employed autologous fascial ligature when urodynamic investigations have revealed that patients presenting with large volumes of SUI exhibit lower maximal urethral closure pressure or ALPP. This is because of the belief that the mechanism of action of the autologous fascial sling is more compressive and, therefore, more suitable for patients with intrinsic sphincter deficiency and more bothersome incontinence. Experts advocate the MUS for women with urethral hypermobility.^8^ The patient herein underwent a repeat sling and showed a high degree of intrinsic sphincter deficiency. Therefore, we decided to perform an autologous fascial sling.

Steven *et al*. analyzed 21 patients for whom synthetic MUSs were ineffective. Patients with a median age of 67 years underwent the procedure. The median postoperative follow‐up duration was 74 months. Before the operation, 57.1% of patients had urethral hypermobility, and 61.9% had mixed urinary incontinence types. Eight patients underwent simultaneous sling excisions, and 76.2% reported successful improvement. Sling excision, mixed urinary incontinence, age at surgery, follow‐up, and initial surgery type were not statistically significant factors. They confirmed that an autologous fascial sling provided reasonable long‐term success as a treatment option.^9^

As TVT or transobturator tape has become the standard technique for SUI, few physicians have experience in autologous rectus abdominis fascia sling surgery. We believe that this method will allow one to reliably perform the fascia sling procedure using the TVT procedure, even if they are unfamiliar with conventional autologous rectus abdominis fascia sling surgery.^8^, ^9^, ^10^, ^11^, ^12^, ^13^

## Author contributions

Yoshitaka Kurano: Data curation; investigation; resources; writing – original draft; writing – review and editing. Nobutaka Shimizu: Data curation; investigation; resources; writing – original draft; writing – review and editing. Rie Yoshimura: Investigation. Ryohei Iga: Resources. Kaya Atagi: Resources. Tomoya Nao: Investigation; resources. Hideo Fukuhara: Resources. Satoshi Fukata: Resources. Shingo Ashida: Resources. Keiji Inoue: Supervision.

## Conflict of interest

The authors declare no conflict of interest.

## Approval of the research protocol by an Institutional Reviewer Board

Not applicable.

## Informed consent

Written informed consent was obtained from the patient.

## Registry and the Registration No. of the study/trial

Not applicable.
